# Advancements in clinical approaches, analytical methods, and smart sampling for LC–MS‐based protein determination from dried matrix spots

**DOI:** 10.1002/jssc.202400061

**Published:** 2024-05-10

**Authors:** Léon Reubsaet, Trine Grønhaug Halvorsen

**Affiliations:** ^1^ Section of Pharmaceutical Chemistry Department of Pharmacy University of Oslo Oslo Norway

**Keywords:** bioactive proteins, dried blood spots, LC–MS determination, smart sampling

## Abstract

Determination of proteins from dried matrix spots using MS is an expanding research area. Mainly, the collected dried matrix sample is whole blood from a finger or heal prick, resulting in dried blood spots. However as other matrices such as plasma, serum, urine, and tear fluid also can be collected in this way, the term dried matrix spot is used as an overarching term. In this review, the focus is on advancements in the field made from 2017 up to 2023. In the first part reviews concerning the subject are discussed. After this, advancements made for clinical purposes are highlighted. Both targeted protein analyses, with and without the use of affinity extractions, as well as untargeted, global proteomic approaches are discussed.

In the last part, both methodological advancements are being reviewed as well as the possibility to integrate sample preparation steps during the sample handling. The focus, of this so‐called smart sampling, is on the incorporation of cell separation, proteolysis, and antibody‐based affinity capture.

**Article Related Abbreviations**
ABC: ammonium bicarbonate
CMC: carboxymethyl cellulose
DBS: dried blood spot
DDA: data‐dependent acquisition
DIA: data‐independent acquisition
DSeS: dried serum spots
DTT: dithiothreitol
DVS: divinyl sulfone
Eu: Europium
Hb: Hemoglobin
hCG: human chorionic gonadotropin
Hct: Hematocrit
HEMA: 2‐hydroxyethyl methacrylate
HR: high resolution
IAA: iodoacetic acid
ICP: inductively coupled plasma
IMER: immobilized‐enzyme reactor
LC–MS: liquid chromatography–mass spectrometry
LcrV: low calcium response V
mAbs: monoclonal antibodies
pHEMA: poly(2‐hydroxyethyl methacrylate)
PRM: parallel reaction monitoring
proGRP: progastrin‐releasing peptide
SDC: sodium deoxycholate
SISCAPA: stable isotope standards by capture of anti‐peptide antibodies
TCEP: tris(2‐carboxyethyl)phosphine
VAMS: volumetric accurate microsampling
VDM: 2‐vinyl‐4,4‐dimethyl azlactone

## 1 INTRODUCTION

Using paper to collect blood samples has a long history, already in the beginning of the 20th century, Ivar Bang described its use for the determination of glucose [1]. Half a century later its potential in neonatal screening was shown by Guthrie and Susi [2] who could detect phenylketonuria in a larger population. It took eight years more to show the ability to determine proteins from dried blood spots (DBS) [3]. In 1986, Wada et al. [4] published the first report on the liquid chromatography–mass spectrometry (LC–MS) determination of proteins from DBS. The hemoglobin variant Yamaguchi was determined using MS. Since that time, many publications on MS‐based determination of proteins from DBS, as well as from other dried biological matrices have been published.

In this review, we will discuss the advances made in the field of LC–MS‐based protein determination from dried matrix spots. The focus will be publications from 2017 and younger and this review will as such be an overview of the advances made in the last years. As can be seen from the discussed publications, the major sample matrix is whole blood collected from a finger or heal prick. However, also other biological matrices such as plasma, serum, urine, and tear fluid can and are collected on paper, hence the term dried matrix spots is used to cover all the different matrices collected. Where appropriate, reference to earlier publications is made. This review is divided into the following parts: first, an overview of reviews is given, followed by an overview of the latest research articles in targeted protein determination and global proteomics for clinical purposes. After this, advances in method development are reviewed followed by advances made in smart sampling. Where appropriate the topics will be clarified by highlighting some typical research reports.

## 2 REVIEWS THAT DISCUSS LC–MS‐BASED DETERMINATION OF PROTEINS FROM DRIED MATRIX SPOTS

Since 2017, several overviews have been published in which the LC–MS‐based determination of proteins from dried matrix samples is discussed. In the sections below these overviews are grouped as follows: general overviews that mention LC–MS‐based protein determination from DBS, reviews on protein determination using MS which mention the use of DBS and reviews on LC–MS‐based protein determination from dried matrix spots.

### 2.1 General overviews of the field of dried blood spots which mention LC–MS‐based protein determination

At least six comprehensive reviews discussing the use of DBS in the determination of low‐ and high‐molecular substances have been published since 2017 [5, 6, 7, 8, 9, 10], all with a different focus. Both Nys et al. [10] and Thangavelu et al. [6] focus on the format of commercially available DBS devices like various types of conventional DBS cards, plasma separation cards, HemaSpot SE devices and volumetric sampling devices like VAMS (volumetric accurate microsampling) and TASSO. This was placed in the context of the main biomedical applications in which they are used: preclinical studies, neonatal studies, animal studies, therapeutic drug monitoring, forensic applications as well as doping analyses and omics applications. Freeman et al. [9] carried out a comprehensive SWOT analysis for DBS analysis within the field of clinical chemistry and reviewed more than 1600 citations and more than 2000 compounds. Among these, many proteins and compounds were measured with MS. These reviews contain limited information on the use of DBS in LC–MS‐based protein determination, but they are very useful for newcomers in the field. Proteins in combination with MS are not specifically mentioned.

The tutorial on VAMS [8] as well as the reviews on multi‐omics from DBS [7] and applications of DBS within clinical chemistry [5] also cover a broad range of compounds but focus either on the use of VAMS [8] or the use of cellulose paper [5, 7] to collect the sample. In the latter reviews, the determination of proteins with respect to the general aspects of targeted and untargeted proteomics from DBS using LC–MS is discussed.

### 2.2 Reviews on protein determination using mass spectrometry which mention the use of dried blood spots

In 2017 the use of DBS for the LC–MS‐based determination of proteins was described as marginal compared with protein determination using samples obtained from conventional sampling [11]. In specific reviews on LC–MS‐based determination of hemoglobin disorders [12] and apolipoproteins [13] the role of DBS samples is discussed. Examples of hemoglobin variant and disorder determination from DBS will be highlighted elsewhere in this paper. The review on apolipoprotein determination focuses on cardiovascular disease risk assessment. It suggests that coupling of LC–MS to remote blood collection could contribute to personalized cardiovascular disease risk assessment as measurement of apolipoprotein from a finger prick monitors the lipid metabolism of patients. This in turn thus contributes to personalized treatment. Remote blood collection also could open for increased sample accessibility as well as allows population‐based research to investigate apolipoprotein‐associated risk factors. Examples of using DBS cards and VAMS devices are mentioned and discussed [13].

### 2.3 Reviews on LC–MS‐based protein determination from dried matrix spots

There is a handful of review articles and perspectives covering specifically LC–MS‐based protein determination from DBS and other dried biological matrices [14, 15, 16, 17, 18, 19, 20]. The reviews of both Nakajima et al. [18] and Lehmann et al. [20] mainly discuss the use of DBS in clinical applications. Together, these reviews are excellent starting points for those who want to develop generic LC–MS‐based methods for the determination of biomarkers or diagnostic proteins from DBS. Various options of sample preparation and analyte enrichment are discussed as well as the right choice depending on the application for data‐independent and data‐dependent acquisition, SRM, MRM, and parallel reaction monitoring all in MS. In their conclusions it is stated that DBS has the potential to become as useful as liquid blood samples, revolutionizing the field of clinical biochemistry [20, 21]. In other reviews [15–17, 19] the horizon is expanded to new frontiers within LC–MS‐based protein determination from DBS. The potential of modifying the sampling device or the material of which the samplers are made is highlighted here. Among this is the concept of smart sampling, which is the integration of sample preparation steps into the sampler. This makes many pretreatment steps, usually carried out at a laboratory before the actual analysis, obsolete. This paper contains a separate section on smart sampling discussing the concept in more detail.

All in all, taking all these reviews together, it shows that the interest in the field of protein determination from DBS is increasing and the application field is expanding rapidly.

The difference between the reviews mentioned above and this paper is that this overview not only covers the latest clinical developments, but it also intends to create awareness that DBS not only is a simple sample carrier: it potentially is a great part of the analytical workflow.

## 3 TARGETED PROTEIN DETERMINATION WITH CLINICAL APPLICATIONS

In the studied literature, mostly all the targeted protein determinations use the bottom‐up approach. In this approach, the disulfide bonds of the target proteins are reduced and alkylated followed by proteolysis, mainly tryptic digestion. This yields proteolytic peptides. The target protein is then determined by means of a target‐protein‐specific peptide: the proteotypic‐ or signature peptide [22, 23]. Depending on the concentration of the target protein in the sample, the determination is either done using affinity clean‐up (in case of low target protein concentrations) or without the use of affinity clean‐up. Below an overview of both approaches is given.

### 3.1 Targeted protein determination with affinity clean‐up

Most of the MS determinations are hampered by the dynamic range: their ability to determine low abundant proteins in the same sample in which high abundant proteins are present. For most MS's the dynamic range is approx. seven orders of magnitude [24]. This number means that it is impossible to measure target protein concentrations lower than approx. 5 ng/mL in a regular blood sample, which contains 35–55 mg/mL albumin (assumed that no pretreatment or depletion of abundant proteins is carried out). In addition to this, matrix effects in the ESI source will have a huge effect on the determination of the target: the presence of peptides from the highly abundant proteins potentially causes ion suppression or enhancement of the target analyte [25]. A way to avoid the above‐mentioned challenges is to perform affinity‐based sample clean‐up. Table 1 shows applications and target proteins measured from DBS and other dried biological matrices. Determination of single proteins as well as multiplexing more than 20 proteins is reported. Additionally, there are mainly two ways to perform affinity clean‐up of the samples. This is either done preproteolytic using monoclonal antibodies (mAbs) to capture the whole target protein or post‐proteolytic using antibodies to capture the proteotypic peptides. The latter is often carried out with co‐extraction of the stable isotopically labeled proteotypic peptide and is referred to as the SISCAPA approach (stable isotope standards with capture by anti‐peptide antibodies).

**TABLE 1:** Applications for targeted protein determination using affinity clean‐up (2017–2023).

Target protein	Application	Singleplex or multiplex (# proteins)	Matrix, sampler material	Extraction preproteolytic/postproteolytic	LOQ	Reference
Pro gastrin releasing peptide	Small‐cell lung cancer	Singleplex	Serum dried serum spots, VAMS	mAb—preproteolytic	1.57 nmol/L^*^	[26]
Transmembrane protein ATPase	Wilson's disease	Singleplex	Whole blood, DBS	SISCAPA—postproteolytic	7.3 – 12.5 pmol/L^*^	[27]
Transmembrane protein ATPase	Wilson's disease	Singleplex	Whole blood, DBS	SISCAPA—postproteolytic	27 pmol/L	[28]
Matrix metalloproteinase 1	Oral cancer	Singleplex	Saliva, dried saliva spots	SISCAPA—postproteolytic	57 pmol/L^*^	[29]
Sotaterecept	Sports—doping analysis	Singleplex	Whole blood, DBS	protein G and Activin A—preproteolytic	11.9 nmol/L^*^	[30]
Acid alpha‐glucosidase, alpha‐L‐Iduronidase	Screening Pompe disease and Mucopolysaccharidosis type I	Multiplex ‐ 2 proteins	whole blood, DBS, buccal swab	SISCAPA—postproteolytic 2 Abs against acid alpha‐glucosidase and 2 Abs against alpha‐L‐Iduronidase	10.2 nmol/L^*^ 18.9 nmol/L^*^	[31]
F1 protein, low‐calcium response V antigen	Biomarkers for Yersinia Pestis	Multiplex ‐ 2 proteins	whole blood, DBS, plasma, dried matrix spots	mAb—preproteolytic	323 nmol/L^*^ (F1 protein)	[32]
CD3e, Wiskott–Aldrich Syndrome protein, Bruton's tyrosine kinase	Severe combined immunodeficiency, Wiskott–Aldrich syndrome, X‐linked agammaglobulinemia	Multiplex ‐ 3 proteins	whole blood, DBS	SISCAPA—postproteolytic	9.9 pmol/L^*^ 19.9 pmol/L^*^ 9.9 pmol/L^*^	[33]
Bevacizumab, trastuzumab, tocilizumab, nivolumab	Therapeutic drug monitoring and Hct determination	Multiplex ‐ 4 proteins	Whole blood, DBS	Protein G—preproteolytic	33.6 nmol/L^*^ 67.1 nmol/L^*^ 87.2 nmol/L^*^ 121 nmol/L^*^	[34]
Wiskott–Aldrich syndrome protein, Bruton's tyrosine kinase, cytochrome b‐245, beta chain, adenosine deaminase, DOCK8, CD42, CD56	Wiskott–Aldrich syndrome, X‐linked agammaglobulinemia, X‐linked Chronic granulomatous disease, adenosine deaminase deficiency, DOCK8 deficiency	Multiplex ‐ 7 proteins	Whole blood, DBS	SISCAPA—postproteolytic	470 pmol/L^*^ 95 pmol/L^*^ 375 pmol/L^*^ 190 pmol/L^*^ 235 pmol/L^*^ 375 pmol/L^*^ 190 pmol/L^*^	[35]
Alpha‐1‐acid glycoprotein; albumin; cystatin C; C‐reactive protein; hemoglobin; haptoglobin; insulin‐like growth factor 1; lipopolysaccharide binding protein; mannose‐binding lectin; myeloperoxidase and serum amyloid A1	Sports—sportomics	Multiplex ‐ 11 proteins	Whole blood, DBS	SISCAPA—postproteolytic	n.r.^**^	[36]
12 inflammation‐related acute phase response proteins	Inflammation, acute phase proteins	Multiplex ‐ 12 proteins	Whole blood, DBS	SISCAPA—postproteolytic	n.r.	[37]
24 clinically important proteins	Establishing personalized protein baselines	Multiplex ‐ 24 proteins	Whole blood, DBS	SISCAPA—postproteolytic	n.r.	[38]

*Note*: Example of estimation for Pro Gastrin Releasing Peptide there is a reported LOD of 6.7 ng/mL [26]. Considering a molecular weight of 14 kDa, a factor of 3.3 between LOD and LOQ (LOQ = 3.3xLOD) and transformation to L, the estimated LOQ is 1.57 nmol/L.

Abbreviations: DSeS, dried serum spots; VAMS, volumetrically accurate microsampling.

* Value is estimated by the authors to make it comparable with other LOQs in this table. This estimation is either on basis of reported LOD and used volume in addition to molecular weight. In case of multiplexing, LOQs are given in the same order as the first column of the table.

** not reported.

The consequence of post‐proteolytic capture is that the digestion needs to be carried out by having the DBS filter paper present. This is not a necessity for applications with pre‐proteolytic capture. One should keep in mind that the effect of this presence should be investigated thoroughly as it might influence the quantitative performance of the method. Additionally, the complexity of the digestion is much higher for postproteolytic capture than with preproteolytic capture. In preproteolytic affinity clean‐up, the target is captured and most of the abundant and unwanted proteins removed before digestion are carried out decreasing the complexity of the sample to a great extent.

Table 1 also shows that there is a relatively large variation in sensitivity: LOQs vary from low pmol/L to high nmol/L range. This should however be seen in relation to the protein targeted, the flow of the LC, and the type of MS used. Either way, it is important to notice that there is a potential for doing sensitive targeted protein determination from dried matrix spots.

An example of a typical preproteolytic affinity clean‐up using mAbs to capture whole target proteins is the multiplex quantification of low‐abundance Yersinia pestis markers in DBSs from mice [32]. In their approach, mAbs against F1 antigen and low‐calcium response V antigen (LcrV‐antigen) are coupled to tosyl‐activated magnetic beads in two different batches. DBSs from mice are first subjected to a sodium deoxycholate (SDC)‐aided protein dissolution from the paper. After removing the paper, the extract was subjected to a generic immunocapture of both the F1 antigen and LcrV‐antigen. This was done by pipetting equal amounts of anti‐F1 and anti‐LcrV beads to the extract. After several washing steps, the beads, with the captured target proteins, were subjected to reduction, alkylation, and digestion. Isotopically labeled peptides (internal standards) are added to investigate the completeness of the tryptic digestion and to correct for variations during injection and analysis. A very informative protocol is provided by the authors (see Figure 2 in [32]). The actual determination was carried out by nano‐LC‐high resolution (HR)MS analysis which was operated in the parallel reaction monitoring (PRM) mode. In this mode, the proteotypic peptides are fragmented and subjected to a high‐resolution full scan in the MS2 and good sensitivity is obtained without the loss of structural information [39]. In this example by Rifflet et al. [32], LODs down to 5 ng/mL were obtained. Besides the fact that DBS is used, this example also highlights its potential in research involving animals. The use of DBS will minimize the need to euthanize the animals to obtain samples.

Similar procedures of pre‐proteolytic affinity clean‐up using mAbs are found elsewhere [26, 30, 34], underlining its generic applicability.

As an example of a typical postproteolytic affinity clean‐up using SISCAPA the work of Razavi et al. [38] is highlighted. They report in a “letter to the editor” the value of longitudinal monitoring of 24 clinically important proteins from DBS. In this way, personal baselines for these proteins could be established. Depending on the protein, this monitoring could be done on a weekly basis while for other proteins denser monitoring frequencies were needed. This report referred to earlier work on how to perform and automate postproteolytic affinity extractions using SISCAPA. Eluate from fixed spots (¼ inch) from the DBS is denatured, reduced, and alkylated followed by tryptic digestion. After termination of the trypsin activity, stable isotopically labelled peptides were added followed by addition of SISCAPA antibodies coupled to magnetic beads to extract the proteotypic peptides. With their approach they can measure both low and high abundant proteins reaching a dynamic range of more than six (a factor of 1,000,000 in difference between the lowest and the highest concentration). Additionally, they present a way to perform normalization for variations in the volume caused by the hematocrit (Hct) effect: through measurement of five proteins (albumin, C3, IgM, plasminogen and hemopexin) a scaling factor is introduced which allows internal normalization of the DBS amount [40]. A similar clinical and identical analytical approach is described by Bassani et al. [36] who studied 11 inflammatory blood proteins in almost 100 athletes over 16 different sports. The clinical similarity in these studies is that longitudinal DBS is carried out to monitor rather general proteins to get insight into the biochemical processes of both patients and athletes. The postproteolytic SISCAPA affinity clean‐up approach is also used in specific disease monitoring by determining disease related biomarkers. Collins et al. [33, 35] describe multiplexing of up to seven specific proteins each directly related to primary immunodeficiency disorders. This shows that the postproteolytic SISCAPA affinity clean‐up is a versatile tool for multiplexing proteins at various concentrations from DBS.

### 3.2 Targeted protein determination without affinity clean‐up

**FIGURE 1:**
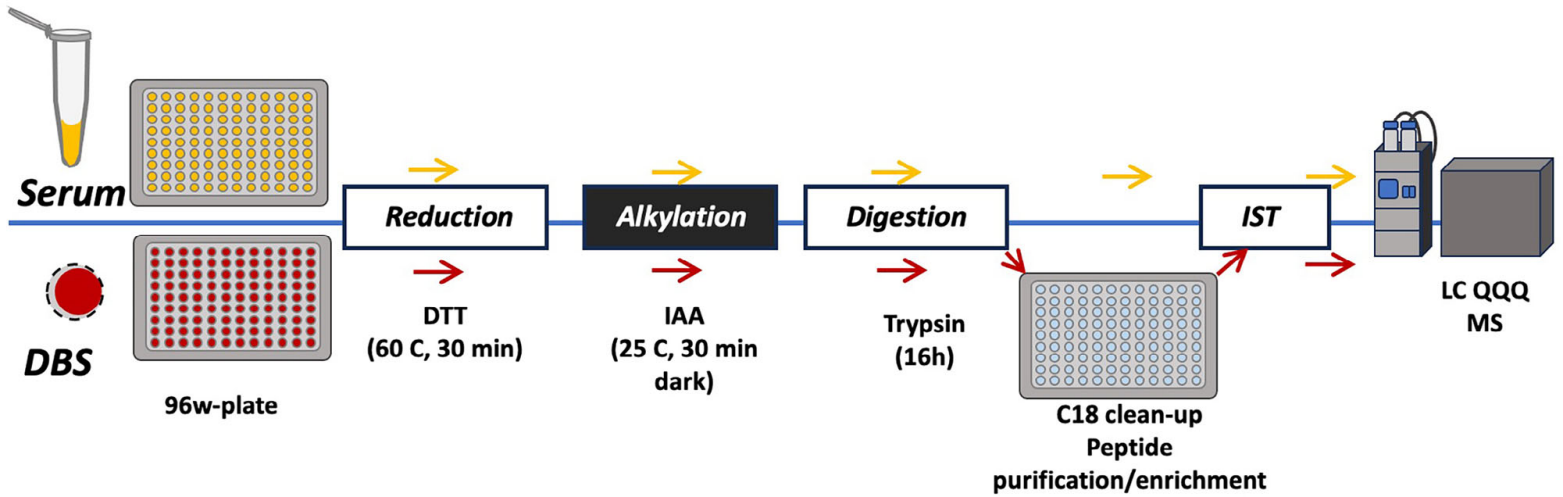
Automated sample preparation for multiplexed targeted protein determination from serum and DBS. The upper part describes the workflow for serum, and the lower part describes the workflow for dried blood spots (DBS). Both workflows contain the same steps. For the DBS workflow an additional C18 clean‐up is used. DTT, dithiothreitol; IAA, iodoacetic acid; IST, internal standard. *Source*: With permission reproduced from Ozcan et al. [41].

When protein determination is carried out without affinity clean‐up, mainly medium and highly abundant targets are measured since these measurements are to a lesser extent hampered by limited dynamic range or matrix effects (see section “Targeted protein determination with affinity clean‐up*”*). There are several examples on multiplexing determinations of medium and high abundant proteins using bottom‐up targeted determination [41, 42, 43, 44, 45]. A typical approach to achieve targeted determination of many proteins was reported by Ozcan et al. [41]. In this study a comparison between workflows using serum and DBS from the same volunteers was carried out. Figure 1 shows the generic workflow used in this specific study. The only difference between the serum workflow and the DBS workflow is the additional extraction and clean‐up: for the latter postproteolytic C18 clean‐up and enrichment was carried out.

The quantification was performed on microbore LC‐QqQ MS/MS monitoring 156 MRM transitions for 156 peptides specific for 82 proteins in total. In general, there was good correlation between DBS and serum for most protein abundances. Only some proteins were more abundant in the DBS samples (like apolipoprotein A‐I and Ig gamma‐1 chain C region) while albumin was more abundant in the serum samples. The authors state that the combination of DBS, which is minimally invasive, with specific and highly sensitive LC–MS/MS operated in the MRM mode allow determination of many proteins in a single run. They conclude therefor that this approach can fundamentally change the field of clinical proteomics and personalized medicine [41]. Targeted determination of hemoglobin (Hb), Hb variants and Hb disorders using LC–MS from DBS has, since the first publication [4], been described often. In the time span focused on in this review only few MS‐based Hb‐specific studies from DBS were reported [46, 47, 48, 49, 50, 51, 52]. Weisinger et at. [47] showed the potential of HRMS for the identification of hemoglobinopathies and β‐thalassemia in DBS. They presented a strategy where a 3.2 mm punch of DBS was extracted for 30 min in presence of trypsin (no reduction and alkylation were performed). After quenching the extract is expected to contain a mixture of both tryptic fragments as well as intact protein subunits present. Analysis was performed using flow injection and Orbitrap HRMS operated in a duplex mode: it scanned in the range m/z 325–1000 for tryptic fragments and in the range m/z 1045 and 1100 for the intact protein. In other words, they integrated the ability to determine intact proteins, which allowed to investigate the intact protein chains α, β, and γ, together with tryptic peptides, which allowed to determine mutations and other hemoglobin related diseases.

**FIGURE 2:**
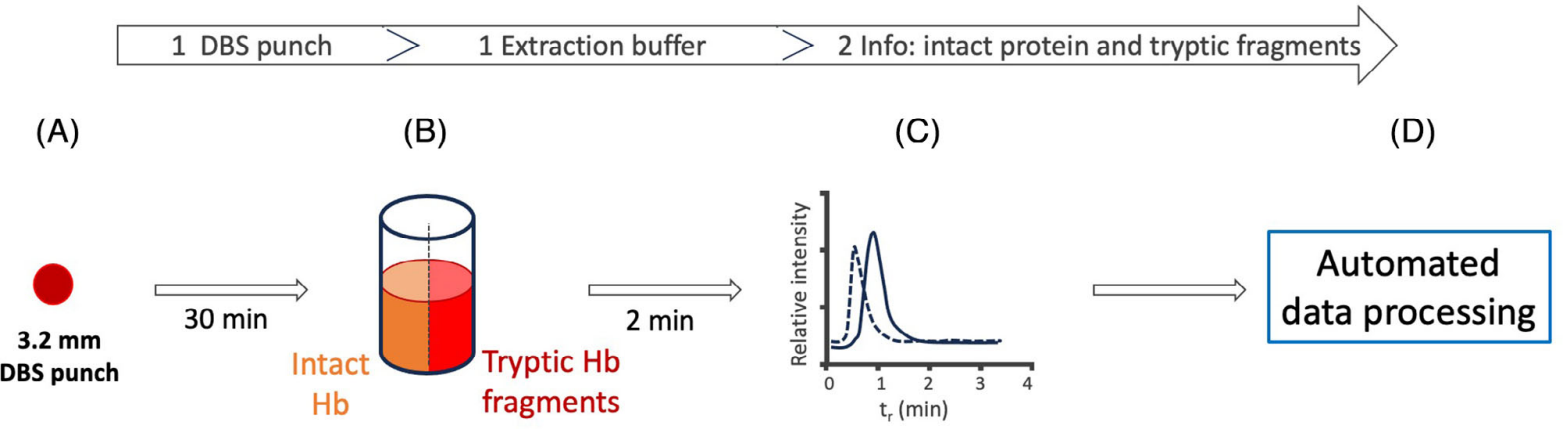
A single dried blood spot (DBS) punch was followed by a short tryptic digestion (A and B). This short digestion leaves some of the proteins still intact but also yields some tryptic peptides. This allowed us to determine both intact protein (—) and tryptic peptides (^___^) in the same sample in a duplex mode after flow injection (C). This was followed by automated data processing using TraceFinder (D). *Source*: Figure adapted after Wiesinger et al. [47].

Within 2 min information on the α, β, and γ subunits as well as on the tryptic fragments for the Hb mutations (Hb C, D, E and S) were obtained (see Figure 2). The authors managed to perform with this experimental set‐up a study on identification of hemoglobinopathies and β‐thalassemia involving DBS samples from 5335 newborns [47].

## 4 GLOBAL PROTEOMIC DETERMINATIONS FROM DRIED BLOOD SPOTS FOR CLINICAL PURPOSES

Not only targeted determinations are carried out successfully, there also have been more publications on global proteomics from DBS for clinical purposes. Since one of the first descriptions on how to determine all proteins in a proteomic experiment from DBS [53], an increasing number of papers have described global proteomic determinations for clinical purposes. The overview of proteomic determinations from DBS (Table 2) shows the use of several approaches. Mainly nano‐LC–MS/MS is used for the instrumental analysis, but also MALDI‐ToF [54] and QqQ coupled to conventional LC [55] has been reported. The latter two approaches by far do not allow as many protein identifications as the nano‐LC–MS/MS approaches.

**TABLE 2:** Applications for global proteomic applications from DBS for clinical purposes (2017–2023).

Application	Sample	procedure highlights—MS tools	Nr. proteins	Reference
Detection of snake envenomation biomarkers	DBS	3 mm disc, extract with SDC, denaturate with urea. Reduction, alkylation, and digestion: TCEP, 2‐chloroacetamide, trypsin. Sample clean‐up and analysis: SPE, nanoLC‐QExactive MS, DDA.	683	[57]
Proteomic analysis of whole blood for precision medicine biomarker studies	VAMS	Most proteins washed away from the tips, then the tips were incubated with buffer, SDC for precipitation. Reduction, alkylation, and digestion: TCEP, chloracetamide, trypsin. Sample clean‐up and analysis: SPE, nanoLC‐HF‐X Orbitrap, DDA, DIA.	Approx. 1600	[56]
Proteomic analysis of patients prior to Takotsubo cardiomyopathy	VAMS	10 μL whole blood—remote sampling. LC–MS, data‐independent acquisition. Principal component analysis (PCA) and t‐test for comparisons.	Approx. 400 to 34 of these gave a difference with normal conditions	[58]
Proteomics‐based detection of Immune Dysfunction in an elite adventure athlete	DBS	4 mm discs. Urea used to denaturate. Reduction, alkylation, and digestion: DTT—iodoacetamide 1:50 ratio trypsin. Digestion for 3 h at 37°C ‐ 1% FA. Sample clean‐up and analysis: SPE C18‐lyophilized—nano‐LC‐MSMS DIA).	712 proteins identified and quantified. 31 upregulated, 35 downregulated	[59]
Clinical proteomics—establishment of a new method for maximal protein count	DBS	3.2 mm discs are placed in a tube. The tube with disc vigorously shaken (5 mm zirconia beads). Add sodium dodecanoate. Supernatant is used further. Reduction, alkylation, and digestion: DTT—iodoacetamide—Trypsin/Lys‐C overnight at 37°C. Sample clean‐up and analysis: C18‐μSPE—nano‐LC–MS/MS (DIA).	1977 proteins	[21]
Clinical proteomics—use a global proteomic approach to make MRM methods for many proteins	DBS	6 mm disc—SDC precipitation—supernatant is used further. Reduction, alkylation, and digestion: TCEP—Iodoacetamide—trypsin (9 h 37°C) ‐ 10% FA (trypsin stop and SDC precipitation)—centrifuge. Sample clean‐up and analysis: SPE—nano‐LC–MS/MS.	>200 proteins	[60]
Clinical proteomics—showcase the use of MALDI‐ToF	DBS	0.25 spot which is subjected to 72 h 0.1% TFA extraction. trypsin o/n 37°C—spotted on plate—dried—α‐cyano‐4‐hydroxycinnamic acid—reflectron, positive ion mode on a ToF MS.	Approx. 30 proteins	[54]
Sportomics—Detection of functional overreaching in endurance athletes	DBS	puncher used—reduction—alkylation and trypsination (no specific information given) Sample clean‐up and analysis: SPE C18 – nano‐LC–MS/MS DIA.	593 proteins	[61]
Schizophrenia risk and urban birth are associated with proteomic changes in neonatal dried blood spots	DBS	3 mm discs Reduction, alkylation, and digestion: DTT—IAA—trypsin 1:20 17 h 37°C. Sample clean‐up and analysis: SPE C18 ‐ QqQ—MRM mode.	Up to 96 proteins	[55]
Proteomic composition dynamics and heart rate variability in dry immersion	DBS	40 μL blood on filter paper—extraction in ABC, SDC, and TCEP—DTT and iodoacetate—trypsin o/n 37°C Sample clean‐up and analysis: SDC precipitation and centrifugation followed by nano‐LC—timsToF.	1256 proteins	[62]

Abbreviations: ABC, ammonium bicarbonate; DBS, dried blood spots; DDA, data‐dependent acquisition; DIA, data‐independent acquisition.; DTT, dithiothreitol; FA, formic acid; IAA, iodoacetic acid; SDC, sodium deoxycholate; TCEP, tris(2‐carboxyethyl) phosphine; VAMS, volumetrically accurate microsampling.

Depending on the sample preparation used and the systems on which the samples are analyzed, up to almost 2000 proteins can be determined from VAMS [56] or DBS [18]. This number becomes even more impressive as one realizes that this is achieved from a single droplet of blood which has been dried. The following descriptions of workflows of proteomic analyses from VAMS and DBS largely show similarities from the point the samples are to be reduced, alkylated, and digested as well as analyzed. The main difference is noticeable for the sample preparation.

Proteomic analysis of whole blood on the VAMS [56] for a high number of protein identifications relied on the removal of most of the protein content from the sampler. In this study, the VAMS tips were washed extensively starting with a 24 h extraction followed by a triple wash using pulse centrifugation. Instead of using the extract or washing solutions, the dry tip was placed in a heated digestion solution (triethylammonium bicarbonate, SDC, tris(2‐carboxyethyl) phosphine (TCEP), chloroacetamide) followed by trypsin digestion at 37°C for 16 h. After digestion termination and SDC precipitation, peptides were desalted using μSPE‐tips. The samples were analyzed by nano‐LC coupled to a HF‐X Orbitrap MS operated in the data‐dependent acquisition (DDA) or data‐independent acquisition (DIA) mode [56].

The method to achieve these large numbers of protein identifications from DBS [18] differentiates from most of the other publications in its sample pretreatment. Nakajima et al. [18] performs two separate extractions: in the so‐called “direct extraction with detergent” the DBS first is disintegrated by vigorously shaking using zirconia beads and subsequent addition of sodium dodecanoate which after several centrifugation steps yields supernatant (extract 1) to be digested. The other extraction relies on both the zirconia beads shaking and sodium carbonate precipitation: sodium carbonate is added to precipitate proteins and here also several centrifugation steps are carried out, this time to yield a protein precipitate. After dissolving, the precipitate (extract 2) is ready for digestion. Both extracts 1 and 2 are digested using dithiothreitol reduction and iodoacetamide alkylation prior to overnight digestion using a mixture of Trypsin/Lys‐C. After C18 SPE nano‐LC–MS/MS using a Orbitrap HRMS operated in the DIA mode was used to maximize protein identifications and combining the information from both extracts [18].

Although it is promising that so many proteins can be determined from a single droplet of sample, it can be stated that for the applications in Table 2, the analytical protocols are both complex and time consuming. The question arises as to what the effect of the protocol used will be on the number and the nature of proteins identified. Additionally, there is a need to explore which proteins and/or tryptic peptides are easily determined from dried matrix sample carriers like cellulose and VAMS. In other words, what is the effect of the dried matrix sample carrier used on the detectability of tryptic peptides.

### 4.1 A note on dried matrix spots analysis in relation to sports

What makes DBS sampling interesting for sports is that it is not as invasive as the conventional blood sampling and therefore easy to perform at the site of athletes. Both determining the effect of physical stress on the athlete's body as well as development and use of methodology to perform doping analyses are application areas for DBS. To get insight into the effect of physical stress Nieman et al. [59, 61] has used global proteomic profiling to detect functional overreaching [61] and immune dysfunction [59] in endurance athletes. In these cases, a bottom‐up approach and nano‐LC coupled to HRMS was used to maximize the amount of information. Besides this, the same researchers also performed profiling of 12 proteins to investigate training distress and illness in swimmers. For this, a bottom‐up approach and LC‐QqQ MS was used for targeted protein determination [63].

Doping analyses using DBS has become attractive and its use was approved by the World Anti‐Doping Agency in the run up to the 2022 Olympic Games in Beijing [64].

Detection of autologous blood transfusions [65], other blood doping practices [66], and abuse of rhEPO through determination of CD71, ferrochelatase, and total erythrocyte protein (Band 3) [67] can be performed using the PRM mode in HRMS after tryptic digestion and nano‐LC separation. These measurements can uncover several types of blood doping all through determination of endogenous proteins. Other proteins for which LC–MS‐based methods from DBS are described are insulin and sotatercept. Determination of both these drugs involved affinity extraction, tryptic digestion (only in case of sotatercept), nano‐LC separation, and HRMS determination [30, 68]. Also, other biological matrices has been investigated for doping purposes: Protti et al. [69] investigated the stability of peptides in urine samples from VAMS in relation to anti‐doping testing of low‐stability peptide hormones and growth factors prohibited by the WADA. All in all, these reports show the growing interest and applicability within the field of sports and anti‐doping testing.

### 4.2 Alternative approaches/improving the workflow of protein determination from dried matrix spots

Besides the clinical advancements made through protein determination from DBS using LC–MS/MS, also analytical advancements were made. In this section these advancements on the sampling material, sample preparation, and analytical instrumentation are discussed shortly.

Regular filter paper (cellulose) is the most used source for sample collection in DBS, however, it also can adsorb proteins [70]. This can also be true for other of the newer sampling materials and devices, and are utilized as an advantage by [56] in their proteomics study. This adsorption can potentially affect the quantitative determination of target proteins negatively as extraction from the sampling material might not be optimal. Rosting et al. [71, 72, 73] addressed this challenge through the introduction of water‐soluble carboxymethyl cellulose (CMC) as sample carrier. As an example of this the application of CMC in the determination of human chorionic gonadotropin (hCG) is highlighted. Whole blood, serum, plasma, and urine were used as sample matrices in the comparison of the performance in hCG determination from regular filter‐paper and CMC. In all cases pre‐proteolytic immunocapture was performed using magnetic beads coated with hCG‐capturing antibodies. The extracts were reduced, alkylated, and digested followed by nano‐LC–MS/MS determination in the SRM mode. Similar LODs were achieved using CMC and regular filter‐paper for whole blood. Slightly better LODs were seen in plasma, serum, and urine using the CMC material. The outcome of real samples did not differ between these materials [71]. It shows the potential of the water‐soluble CMC as a sample carrier, but more research is needed to make it a viable alternative to cellulose.

Wouters at el. [74] addressed the challenge of the time consuming trypsinization needed for bottom‐up determination of proteins. They developed a cyclic‐olefin‐copolymer microfluidic immobilized‐enzyme reactor (IMER) with trypsin as the proteolytic enzyme. This allowed rapid digestion from DBS extracts. The flow‐through of the IMER was analyzed using nano‐LC–MS (triple ToF in the information dependent mode). Already with very short residence times in the IMER (<1 min) high sequence coverage was obtained. Without the use of reduction and alkylation steps similar numbers of proteins were identified in a proteomic experiment (146 proteins) compared with a conventional in solution digestion of the DBS extract (154 proteins). The challenge of this time‐consuming trypsin digestion from DBS is addressed also in the concept of smart sampling which is being discussed later in this review.

From an instrumental viewpoint most of the analyses are carried out using (nano‐) LC–MS/MS. To our knowledge, the use of IMS in protein determination from DBS is scarcely described. Rosting et al. [75] coupled automated liquid surface extraction to extract tryptic peptides from DBS to field asymmetric waveform ion mobility spectrometry greatly improving the number of peptides and proteins identified from the sample. Thomas and Thevis [68] successfully combined conventional LC with IMS and HRMS allowing them to determine and differentiate between endogenous human insulin and synthetic analogs Aspart, Detemir, Glargine, Glulisine, Lispro, and Tresiba. Although insulin has a mass of approx. 6000 Da, no tryptic digestion was carried out. Differences in retention time and drift time together with good resolution and sensitivity of HRMS made differentiation possible. Both studies show the potential of introducing an additional separation dimension in the workflow of protein determination from DBS cards.

**FIGURE 3:**
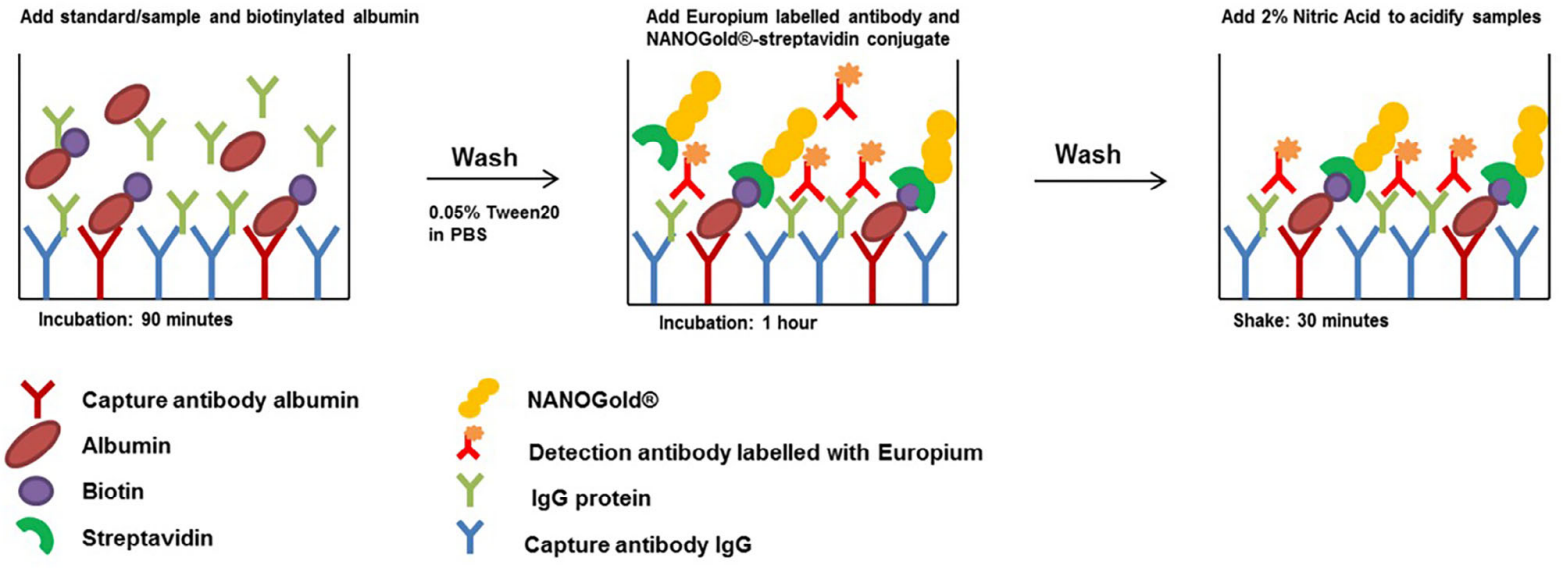
Schematic representation of sample preparation steps prior to simultaneous determination of albumin and IgG by ICP‐MS through respectively NANOGOLD and Europium (Eu). In the first step, both albumin and IgG are captured by their respective antibodies. After a wash, NANOGOLD coupled to streptavidin (to detect albumin) and antibodies labeled with Eu (to detect IgG) are added. ICP–MS was carried out after removal of residual reagents. *Source*: With permission reproduced from Hogeling et al. [76].

Hogeling et al. [76] designed a strategy for the targeted determination of proteins from DBS using inductively coupled plasma–MS (ICP–MS). ICP‐MS is not a logical choice as it typically is used in elemental analysis. In this study, ELISA was performed using wells coated with anti‐albumin and anti‐IgG antibodies to capture albumin and IgG from a DBS extract. For the albumin a competitive approach was chosen. To the sample biotinylated albumin was added (competitive assay) which in its turn was sandwiched using NANOGOLD®‐tagged streptavidin. The captured IgG was sandwiched using Europium (Eu)‐labeled antibodies. After a washing step to remove the excess of NANOGOLD® and Eu, ICP–MS was carried out on the content of the wells (Figure 3).

The signal of gold inversely correlated with the albumin concentration while the signal of Eu directly correlated with the IgG concentration. Comparable results for albumin and IgG were obtained for ELISA and ICP‐MS from DBS. This thus represents a novel proof of concept study for simultaneous quantification of proteins in DBS. It is, with the already existing techniques for proteomic analysis, not very likely that there will be many ICP‐MS applications within the field of MS‐based protein determination from dried matrix samples.

The use of paper spray for protein determination from DBS is also described to a low extent. There were promising reports on the determination of low molecular substances from DBS [77, 78]. However, for the determination of proteins from filter‐paper, only a single publication is known to the authors: Li et al. [79] modified cellulose with polystyrene for the successful top‐down determination of model peptides and proteins (angiotensin II, cytochrome c, hemoglobin, lysozyme, and myoglobin) from whole blood. Although the concept of paper spray is proven, development and applicability for MS‐based protein determination from dried matrix samples still is in its infancy.

## 5 ADVANCES IN SMART SAMPLING

Many of the applications discussed above require extensive sample preparation to make the sample compatible for high end LC–MS analysis. These, in some cases time consuming, steps are to be carried out when the dried sample arrives at the laboratory, thus contributing to long response times. Efforts to speed up this process after arrival in the laboratory have been described above: both IMERs [74] and paper‐spray [77, 78] have shown to be promising in shortening the time of sample preparation. There are, in addition, several efforts to speed up this process by integrating parts of the sample preparation in the sampling device. In this way the sampling device becomes an active part in the sample preparation. This so‐called smart sampling has the advantage that no changes in sampling protocols are needed for the patient, keeping the comfort of sampling at home. The largest change for the analytical side (laboratory) is that the workflow can be simplified since preanalytical steps already are carried out on the smart sampler. Below the latest advances in smart sampling are discussed.

### 5.1 Cell separation on‐card

**FIGURE 4:**
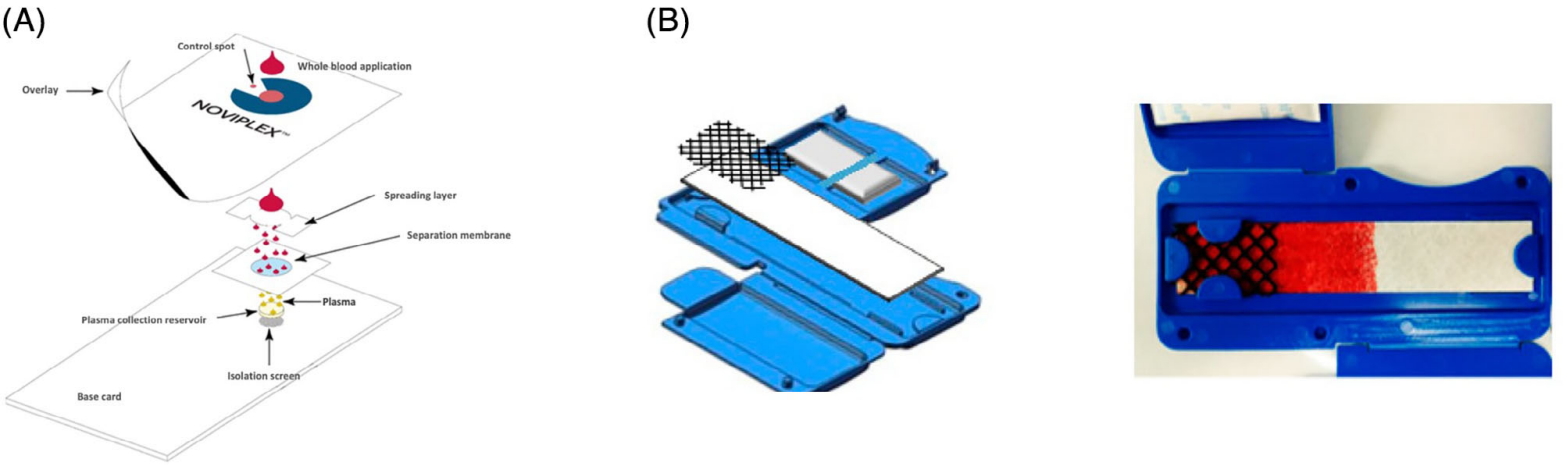
(A) Noviplex cell separation card. *Source*: With permission reproduced from Kim et al. [82]. (B) Cell separation based on lateral flow. Both devices separate blood cells from plasma. *Source*: With permission reproduced from Kaiser et al. [85].

Collection of whole blood on filter‐paper DBS cards causes lysis of blood cells [80, 81]. This releases intracellular proteins which in specific cases might be unwanted or disturbing to the analysis. Separation of blood cells from plasma in conventional liquid blood samples is done by centrifugation but is not possible with conventional filter‐paper DBS cards. Kim et al. [82] presented in 2013 the first DBS card which had an integrated blood cell/plasma separation for proteomic purposes. The separation between blood cells and plasma is facilitated by a separation membrane principle (see Figure 4A). This principle was applied in several clinical studies. A comparative study between proteomic data derived from fresh‐frozen serum samples and serum dried from blood deposited on Noviplex cards for patients with intrauterine growth restriction was carried out by Wölter et al. [83]. They show that the serum protein profiles for from both sample types were of comparable quality. Pollard et al. [84] reported on nonlethal blood sampling of fish in the lab using Noviplex cards with the purpose of LC–MS‐based proteomic analyses. A different blood sampling device with integrated cell separation and intended for downstream proteomic analysis, was presented by Kaiser et al. Figure 4B shows its design. In this design blood (250 μL) is deposited on the upper part of a membrane. The mesh in this part facilitates evenly lateral flow of the sample which will distribute throughout the device; however, blood cells have more retention and will travel approximately half ways. The remaining, liquid part of the whole blood sample will reach the end of the device. In this way cell separation is achieved. The plasma portion was in this study, used for both top‐down protein determination by MALDI‐ToF and bottom‐up protein determination on an LC‐QqQ MS/MS operated in the MRM mode [85].

Although in the above‐mentioned cases, the devices were used in a controlled setting, it shows that cell separation of whole blood can be integrated during sampling for LC–MS‐based protein determination from plasma.

### 5.2 Tryptic digestion during sample collection: Smart proteolytic sampling

**FIGURE 5:**
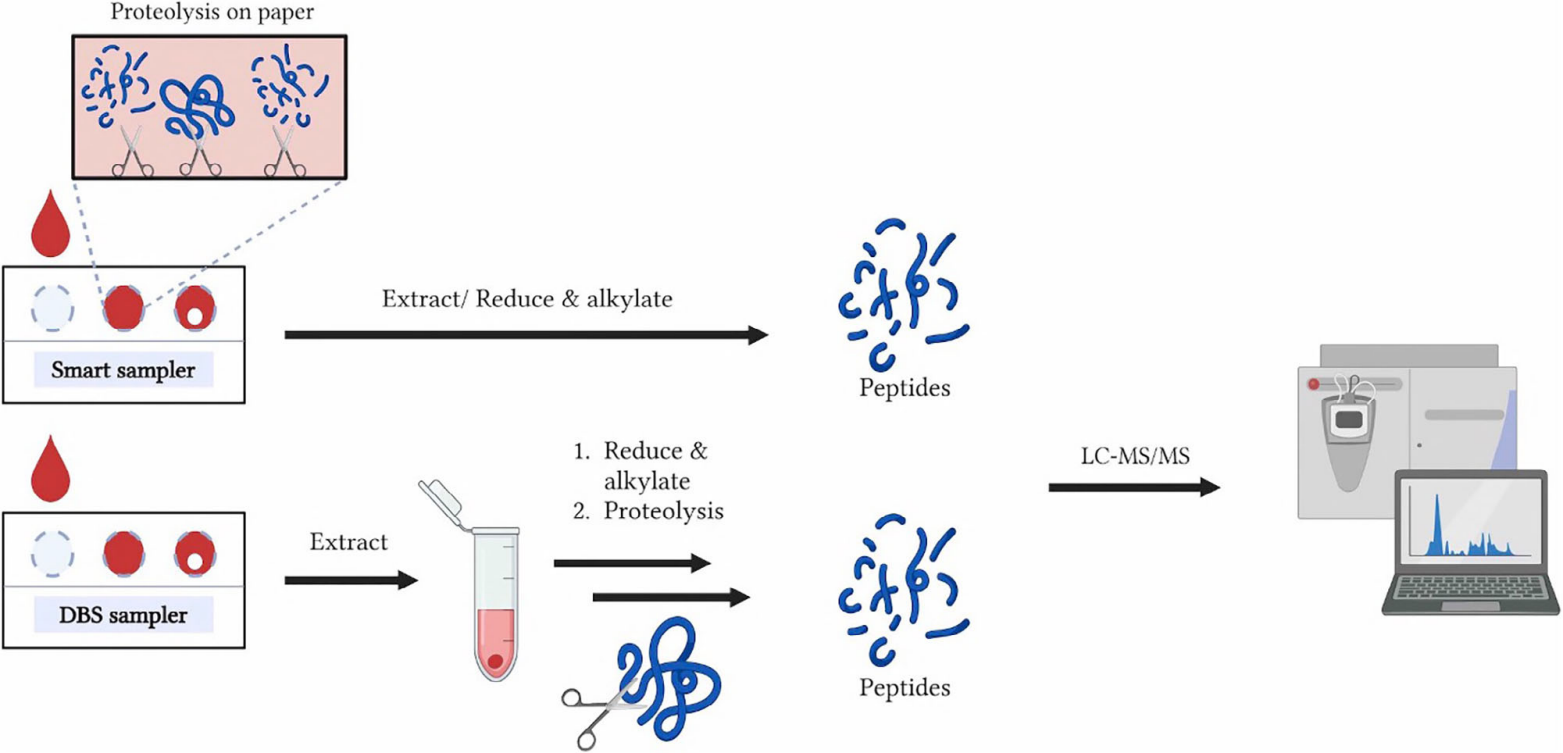
Comparison of Smart proteolytic sampling—these samplers exhibit tryptic activity (upper part) with conventional workflow (lower part) for bottom‐up determination of proteins from DBS. *Source*: With permission reproduced from Johannsen et al. [16].

The most time‐consuming step in the bottom‐up determination of proteins is the enzymatic digestion. Integration of this sample preparation step potentially can save a lot of time. Integration of trypsination with the purpose of LC–MS‐based protein determination from dried blood on paper was first described by Skjærvø et al. [86]. The concept of smart proteolytic sampling is based on dripping the sample on filter‐paper which has proteolytic activity. During drying at ambient temperature, the proteins in the sample are digested and after extraction ready for LC–MS analysis. A comparison between smart proteolytic sampling and conventional bottom‐up determination is shown in Figure 5. It should be noted that one of the differences between the digestion using smart proteolytic sampling and conventional digestion, is the order in which reduction and alkylation is carried out: for the conventional digestion, this is carried out before proteolysis, while in the smart proteolytic sampling, it is carried out after the proteolysis. This means that for the smart proteolytic samplers, proteins still contain disulfide bridges.

In the first study [86] Sepharose beads immobilized with trypsin were deposited on paper on the inside of a wax printed circle. The wax was used to keep the blood sample within the boundaries of the circle. In a follow‐up study, trypsin was covalently bound to cellulose. This was done through functionalization of the cellulose, followed by the immobilization of trypsin. The functionalization was done using poly (2‐hydroxyethyl methacrylate) 2‐vinyl‐4,4‐dimethyl azlactone (pHEMA‐VDM) chemistry: first the cellulose fibers were silanized before being activated in two steps before immobilizing trypsin. Although successful in digesting proteins from whole blood, the samplers did not absorb blood to the same extent as untreated cellulose [87]. This was seen as disadvantageous and addressed in a subsequent study through a less laborious functionalization (direct oxidation of cellulose through periodate [88, 89, 90]) leading to less substantial changes in the cellulose. This simple one step functionalization allowed to immobilize trypsin retaining up to 60% of its activity compared with in‐solution digestions [90]. These smart proteolytic samplers were stable for at least 4 months at 4°C additionally showing little autolysis [86, 90]. Trypsin immobilization was not only carried out on cellulose but also on VAMS microsampling devices [91]. These commercially available polymeric samplers were in this manner transformed from sample carriers to sampling devices that carried out proteolysis instantaneously.

Smart proteolytic sampling has been applied in global proteomic approaches from whole blood [86, 87] and from serum [89, 90, 91]. Also use in targeted protein determination is described [92]. In this study, progastrin‐releasing peptide (proGRP) was used as a model protein to be determined from serum. As little as 15 μL of serum was dripped on the smart proteolytic sampler and left to dry at ambient temperature. After this, the proteolytic peptides were extracted in the presence of anti‐proGRP mAbs coupled to magnetic beads. In this manner, the signature peptide ALGNQQPSWDSEDSSNFK was captured and analyzed using a nano‐LC‐QqQ MS operated in the SRM mode. A LOD down to 150 pg/mL was achieved, showing the applicability of smart proteolytic samplers to determine very low abundant proteins [92].

Although the concept of integrating trypsin is promising, there still is a need for extensive further investigations before it can be used for routine applications: parameters like analytical reproducibility and completeness of the proteolysis need more attention to be able to perform quantitative determinations.

### 5.3 Target protein capture during sample collection: Smart affinity sampling

**FIGURE 6:**
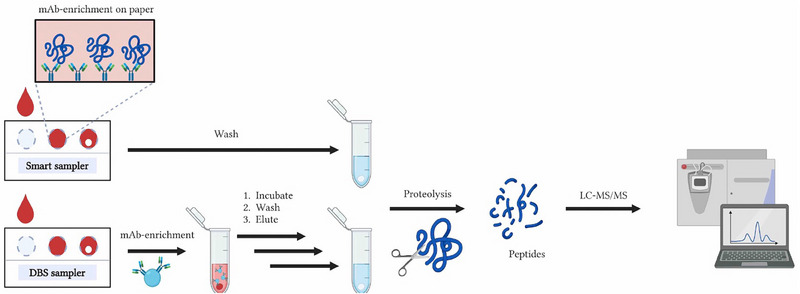
Comparison between smart affinity sampling—these samplers have immobilized mAbs on the surface to capture target proteins (upper part) and the conventional workflow (lower part) for targeted protein determination from DBS. *Source*: With permission reproduced from Johannsen et al. [16].

Integration of target protein capture on filter‐paper for the LC–MS‐based determination of low abundant proteins was first published in 2019. Skjærvø et al. [93] used three strategies to immobilize anti‐hCG antibodies to cellulose: via adsorption, after pHEMA‐Tosyl functionalization and after pHEMA‐VDM functionalization, the latter being the most promising. The analytical workflow to determine hCG in serum was as follows: serum containing hCG was dripped on the smart affinity sampler and left to dry. After a wash procedure including various buffers and Tween to remove most of the serum proteins, reduction, alkylation, and digestion was carried out. The sample was then directly injected into a nano‐LC‐QqQ MS for analysis [93]. Figure 6 shows a comparison between smart affinity sampling and the conventional workflow for targeted protein determination from DBS.

**FIGURE 7:**
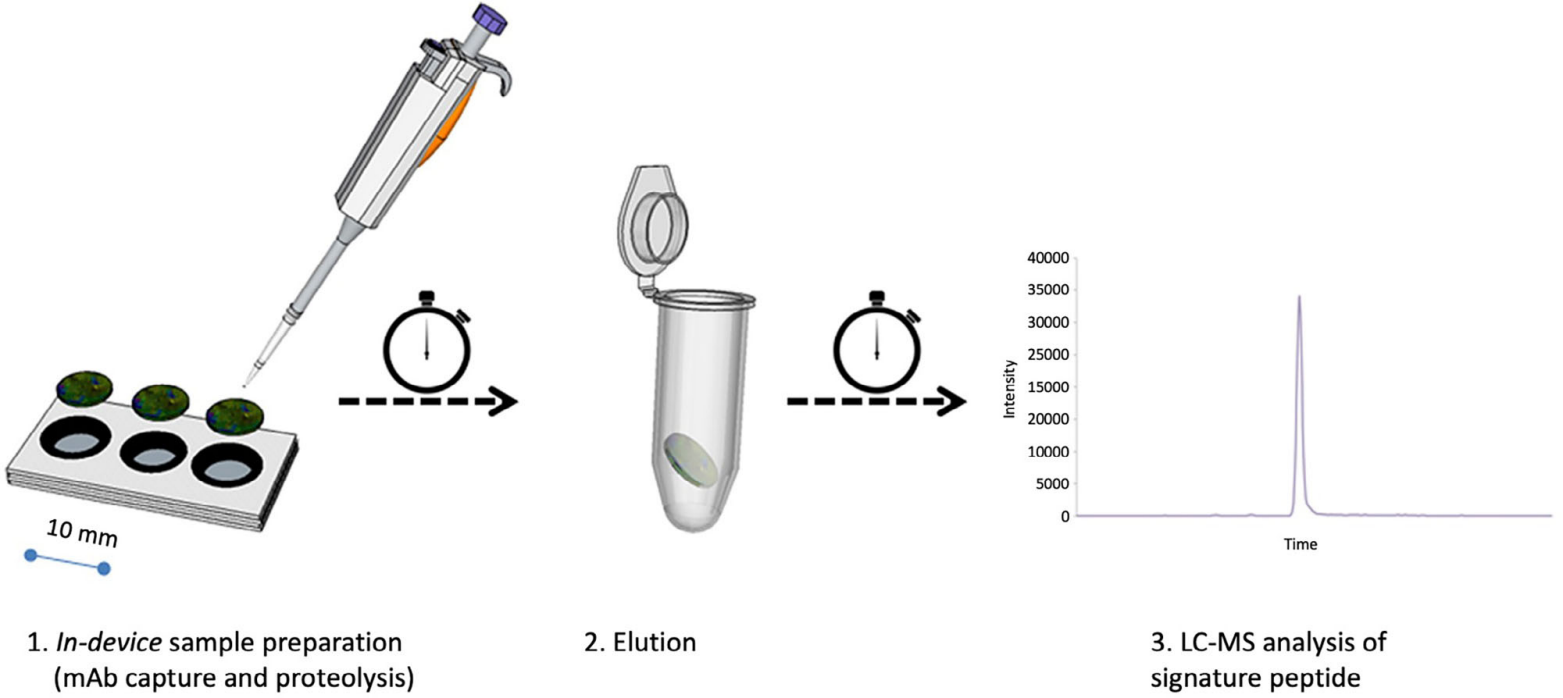
The device consisted of four layers of paper. The bottom layer was covered completely with wax. The following three layers were stacked. Each layer contained wax‐printed circles of which the inside was punched out creating a paper well. The paper reactor was placed in this paper well and all reactions were carried out in the same well. *Source*: With permission reproduced from Skjærvø et al. [94].

The smart affinity sampling concept was developed further to a device consisting of three layers of filter‐paper with holes and a bottom layer of filter paper. This resulted in a paper device with small wells in which the paper disc with immobilized mAb was placed (see Figure 7). Here, capture of hCG was carried out followed by washing, reduction, alkylation, and digestion before injection into the nano‐LC‐QqQ MS operated in the SRM mode for analysis.

Both serum and whole blood could be analyzed, and the time‐use of the whole analytical procedure was reduced by a factor of six. The LOD for dried serum (100 pg/mL) and for dried whole blood (LOD of 630 pg/mL) [94] again showed the potential for low abundant protein determination using smart samplers.

Besides adsorption, pHEMA‐Tosyl and pHEMA‐VDM also other strategies to immobilize mAbs were investigated not the least to improve the absorbing properties of the functionalized cellulose. The use of divinyl sulfone (DVS) to functionalize filter‐paper [95] has been described by Mrsa et al. [96]. This low labor‐intensive functionalization which, after immobilization of the mAb, leads to a smart affinity sampler with the same absorbing properties as untreated filter paper and similar performance as the above‐described pHEMA‐VDM sampler, makes DVS a viable alternative to functionalize cellulose.

Also, the earlier described periodate oxidation has been investigated for the immobilization of antibodies. Johannsen et al. [97] designed a smart affinity sampler by functionalization of the cellulose through periodate oxidation followed by immobilization of streptavidin. Streptavidin does not capture proteins or antibodies; however, it serves as an anchor for biotinylated mAbs. Because of the strong interaction between streptavidin and biotin only a relatively short and simple incubation with biotinylated mAbs is necessary to produce the smart affinity sampler. In this specific report the proof of principle was shown using hCG as a model compound. It is concluded that this concept not only can be used for hCG determination, it could also readily be used for other biotinylated mAbs as suggested by [97].

Even though applicability of smart affinity samplers is proven for real samples, there is the need to confirm their quantitative potential. This can be done through full validation of the samplers for use in realistic applications according to FDA‐ or EMA‐guidelines. Benchmarking with existing analytical methods will be essential to investigate if they have an added value and if it can be added to the toolbox of the analytical‐ and clinical chemist.

## 6 CONCLUSION

As seen from the number of publications focussing on MS‐based determination of proteins from dried matrix spots, the field is expanding rapidly.

From a clinical point of view, the targeted determination of biologically relevant proteins which are low abundant immuno‐capture is needed. Applications ranging from the determination of a single protein to the multiplexing of 24 proteins are reported. For the targeted determination of biologically relevant proteins in the medium and high abundance levels no additional immuno‐capture is needed. In specific, determination of hemoglobin and its variants still is a protein in research focus. In addition to this multiplexing up to 82 proteins simultaneously is described. For the global proteomic approach considerable improvements have been made allowing identification of up to almost 2000 proteins from a single droplet of dried blood.

From a methodological point of view, the use of IMS shows to be a technical advancement which enabled increased peptide and protein identifications from DBS. Although mainly HRMS and QqQ MS are used for quantitative purposes in general, the use of ICP–MS was introduced for targeted protein quantification.

Advancements in the sample preparation was mainly integrating sample preparation during sample handling. This is defined as smart sampling. The three main preparation steps integrated are cell separation, proteolysis, and immunocapture. For the latter two it has become possible to modify cellulose in such a way that it can perform both tryptic digestion as well as antibody‐based affinity extractions without loss of the absorbing properties of filter‐paper. Both global proteomic determinations, as well as targeted quantification (down to sub ng/mL level), are possible.

As the trend of patient centric sampling still is in its infancy, we believe that within the coming years, DBS will be implemented in routine clinical analysis of an increasing number of protein biomarkers and drugs. The general trend in implementing automated workflows for DBS handling and protein sample preparation will contribute to this growth. Collection of dried matrices for simple storage of small volume biobank samples is also expected to expand, making dried matrix spot sampling relevant for future proteomics studies. Simplification of the sample preparation workflow after arrival of protein samples to the laboratory through smart sampling is also expected to contribute to this trend.
